# Do men perform better than women in trauma?

**DOI:** 10.1186/cc13748

**Published:** 2014-02-27

**Authors:** Alberto Hernández-Tejedor, Carlos García-Fuentes, Emilio Alted-López

**Affiliations:** 1 Unidad de Cuidados Críticos, Hospital Universitario Fundación Alcorcón, C/. Budapest, 1, 28922 Alcorcón (Madrid), Spain; 2 Unidad de Cuidados Intensivos, Hospital Universitario, 12 de Octubre Av, Córdoba s/n. 28041 Madrid, Spain

## Abstract

In recent decades, numerous studies have compared survival according to gender of patients admitted to general hospitals and particularly to intensive care units. In a previous issue of *Critical Care,* Schoeneberg and colleagues presented the results of a German observational study on a sample from a 10 year registry in a Level 1 trauma center. The conclusion is that there is a trend towards a higher mortality in women than in men.

## 

Epidemiological studies provide insight into certain features of diseases that may be useful when developing preventative or treatment strategies. Not only trauma but many other diseases have been studied, comparing and analyzing the different course in men and women, giving rise to various hypotheses. In a previous issue of *Critical Care,* Schoeneberg and colleagues [1] report their results of a study comparing outcome according to gender of severe trauma patients admitted to a Level 1 trauma center in Germany.

Firstly, we should appreciate that the prevalence of different injury mechanisms can be different in males and females, with a higher frequency of stab and traffic accident injuries in males. The injury pattern is different according to gender since most injuries occur in environments and circumstances that show differences in behavior between men and women, such as leisure, workplace or the use of motor vehicles [2]. Also, as expected, differences in mortality between men and women become smaller when only patients with very high Injury Severity Scores are considered, as mortality is usually inevitable in these extremely serious injuries.

Schoeneberg and colleagues [1] have conducted numerous comparisons, increasing the likelihood that some of them show a statistically significant result. In particular, absolute volumes of fluid replacement administered are lower in women than in men, but women usually have a lower body weight, so that the volume administered per body weight may not be different. Hemoglobin concentration at admission is also lower in women than men, although this may not reflect a more severe hemorrhage but a lower baseline value (hemoglobin concentration is typically lower in healthy women than men, mainly in childbearing age). Although the analysis shows a trend towards a higher mortality in women in most of the analyzed subgroups, when potential confounders are adjusted for, such as age or Abbreviated Injury Scale, these differences are no longer statistically significant.

Gender comparisons in general populations of critically ill patients have come to divergent conclusions, but improved survival in women probably predominates [3,4]. These observations have led to numerous hypotheses, usually around hormonal differences or immune issues [5,6]. In the case of multiple trauma patients, similar studies have been performed but most have concluded that overall mortality is not influenced by gender [7,8]. These studies are usually single-institution studies, so conclusions cannot be drawn about population genotypic differences that may lead to different results depending, for example, on race.

In an attempt to find a practical reason for the observed differences, animal studies have been conducted and suggest that female sex hormones can be neuroprotective. In fact, several studies document improved neuronal survival with administration of estrogen or progesterone to males or ovariectomized females [9,10]. However, exogenous administration of sex hormones may not replicate the natural effects - and assumed benefits - of the menstrual cycle.

One cannot conclude that there is definitely higher mortality in injured women than men, although there is such a tendency. Other factors may mitigate the influence of gender in trauma.

## Competing interests

The authors declare that they have no competing interests.
